# Heyde’s Syndrome Manifesting as Recurrent Gastrointestinal Bleeding After Valve Replacement

**DOI:** 10.7759/cureus.75500

**Published:** 2024-12-10

**Authors:** Deborah L Núñez, Adela G Solis Lopez, Paula M Cuéllar Pinzón

**Affiliations:** 1 Internal Medicine, Hospital Universitario Dr. José Eleuterio González, Monterrey, MEX

**Keywords:** aortic stenosis (as), gastrointestinal tract bleeding, heyde’s syndrome, intestinal angiodysplasia, von willebrand diseases

## Abstract

Heyde’s syndrome is a clinical entity that combines aortic stenosis, gastrointestinal angiodysplasia, and an acquired von Willebrand factor disorder. This syndrome is characterized by the association between aortic stenosis and recurrent gastrointestinal bleeding episodes, typically linked to angiodysplasias. Effective treatment requires addressing the underlying condition, specifically aortic stenosis, which leads to the structural destruction of coagulation proteins, resulting in the acquired von Willebrand factor disorder and perpetuating the bleeding. Therefore, managing gastrointestinal bleeding alone is insufficient. Although initially underestimated by physicians due to its nonspecific presentation and overlapping symptoms, this syndrome has significant implications for diagnosis and management, particularly in older adults.

Many patients with Heyde’s Syndrome are often misdiagnosed with unrelated gastrointestinal conditions until the association with aortic stenosis is identified. This diagnostic delay can lead to repeated hospitalizations, chronic anemia, and a decline in quality of life. Aortic valve pathology and coagulopathy should be actively suspected and investigated, directing treatment toward correcting the aortic stenosis.

The objective of this case report is to highlight the importance of suspecting this syndrome in patients with valvular disease, such as aortic stenosis, and recurrent bleeding episodes, as these conditions may not always represent two independent problems, even if the patient has previously undergone valve replacement. This is demonstrated in the presented case, where a 74-year-old female patient with cardiovascular disease treated years earlier with valve replacement developed valve dysfunction, leading to new episodes of gastrointestinal bleeding. This illustrates the need to reevaluate the valve to prevent recurrent complications.

## Introduction

Heyde’s syndrome is a systemic condition characterized by the association of aortic stenosis, gastrointestinal angiodysplasia with recurrent gastrointestinal bleeding, and an acquired von Willebrand factor disorder [1]. This condition was first described in 1958 by Dr. Edward C. Heyde, who noted a relationship between aortic stenosis and gastrointestinal bleeding, generally due to angiodysplasia. Although initially thought to be a coincidence in older adults, studies have demonstrated a real link attributable to the loss of von Willebrand factor multimers. Shear stress in conditions such as aortic stenosis unfolds the von Willebrand factor multimers, exposing their A2 domain, which allows the enzyme ADAMTS13 to cleave them, reducing the high-molecular-weight multimers essential for platelet adhesion. As a result, an acquired von Willebrand syndrome (type 2A) develops, increasing the risk of bleeding, particularly in areas of high hemodynamic stress, such as gastrointestinal angiodysplasias [2].

In the literature, the prevalence of Heyde’s syndrome is reported with two separate figures: 1% in the general population in the United States and 2%-10% in patients with concurrent angiodysplasia and aortic stenosis [3]. This apparent discrepancy may be explained by differences in the populations studied or significant underdiagnosis. While the 1% reflects an estimate based on the general population, the 2%-10% range likely represents a higher prevalence in studies focusing on patients with more specific clinical characteristics, such as the active coexistence of angiodysplasia and aortic stenosis.

Understanding this prevalence not only highlights the need for precise diagnosis but also underscores the clinical relevance of Heyde’s syndrome. This disorder has profound implications for cardiovascular and gastrointestinal pathophysiology, directly affecting clinical management and patient outcomes. Its timely identification and treatment can prevent recurrent hospitalizations, improve quality of life, and reduce morbidity associated with chronic anemia and recurrent bleeding episodes.

## Case presentation

A 74-year-old female patient with a history of aortic stenosis treated with a biological valve replacement eight years ago, which had been dysfunctional for the past five months, was under evaluation for a repeat valve replacement. She had been hospitalized five times over the last three years due to episodes of upper gastrointestinal bleeding. These hospitalizations had identified anemia without transfusion requirements and endoscopic findings of peptic ulcer disease and diverticular disease as incidental findings, with no active bleeding observed.

The patient presented to the hospital after experiencing three episodes of hematochezia, along with diaphoresis, asthenia, and adynamia over two days.

Upon arrival at the emergency department, she was somnolent, with a Glasgow Coma Scale (GCS) score of 13, blood pressure of 80/50 mmHg, heart rate of 123 bpm, a capillary refill time of three seconds, no respiratory compromise, and was afebrile. Initial resuscitation included the administration of 1000 mL of lactated Ringer’s solution as a bolus, which stabilized her hemodynamic status (blood pressure 110/60 mmHg, capillary refill time of two seconds). This was followed by a continuous infusion of 1000 mL of lactated Ringer’s solution at 83 mL/hour. Additionally, a single dose of 80 mg of intravenous omeprazole was administered, followed by 40 mg every 12 hours.

Initial laboratory results showed microcytic hypochromic anemia (Hb 8.30 g/dL, MCV 68 fL, MCH 26 pg), while blood chemistry, electrolytes, and liver function tests were within normal limits. During her stay in the emergency department, the patient remained under continuous cardiac monitoring. After six hours of observation with no signs of hemodynamic instability, she was admitted to the Internal Medicine department for further evaluation. A gastroenterology consultation was requested for endoscopy and colonoscopy.

**Figure 1:**
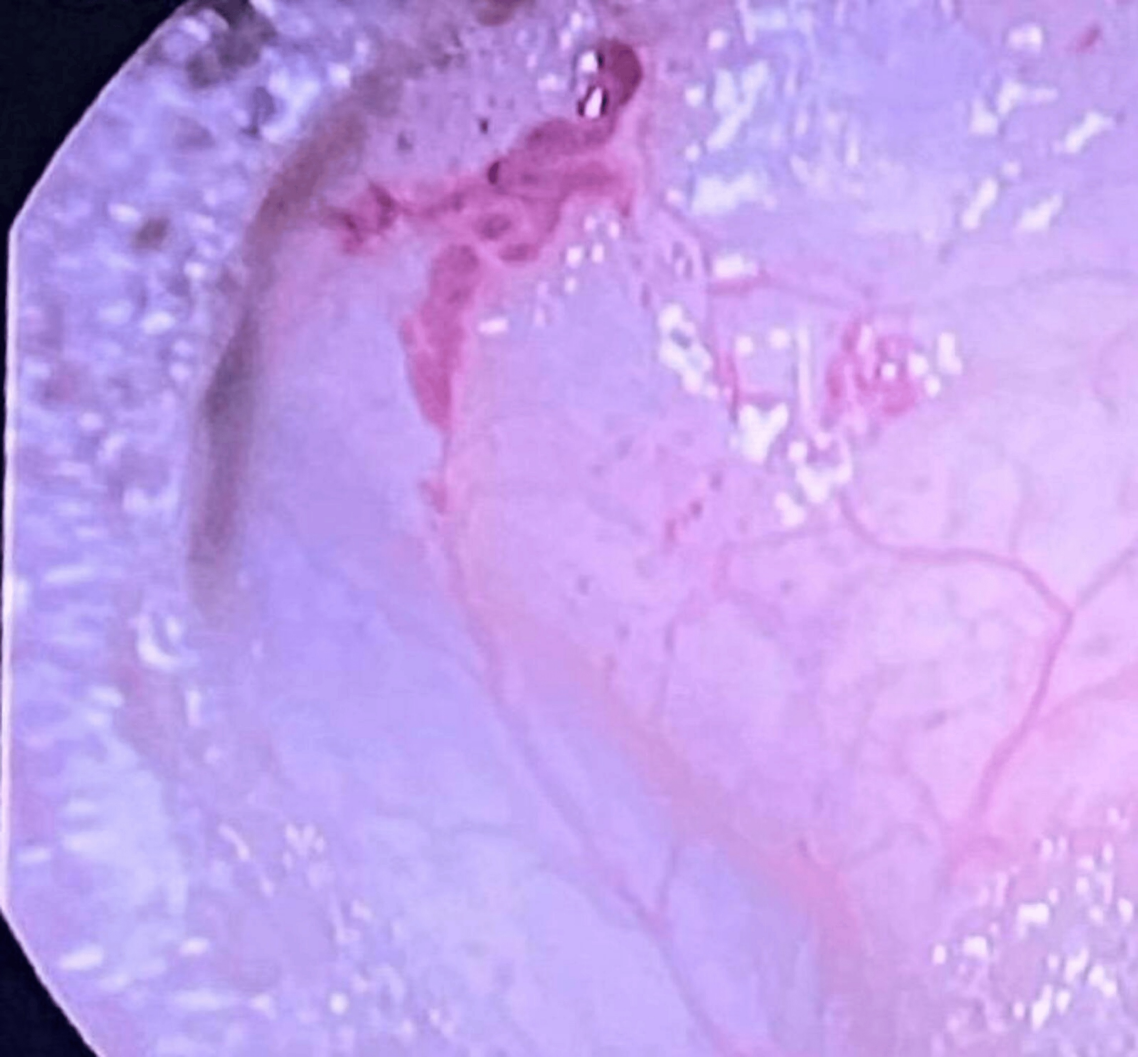
Endoscopy with angiodysplasia in the greater curvature of stomach.

Endoscopy revealed a type I hiatal hernia, atrophic and erosive gastritis in the antrum, a Forrest 3 gastric ulcer, and angiodysplasia in the greater curvature (Figure 1). Colonoscopy showed pan-diverticulosis and uncomplicated external hemorrhoids, with no evidence of active bleeding.

The identification of gastric angiodysplasia was key and led to an in-depth review of the literature to investigate its potential correlation with aortic stenosis, which ultimately raised suspicion for a diagnosis of Heyde’s syndrome. A targeted approach was taken, and a von Willebrand factor profile was requested, which revealed a decrease in ristocetin cofactor activity (VWF:RCO) to 25% and the absence of high-molecular-weight multimers. This decrease indicates an issue with von Willebrand factor (vWF), a key protein in coagulation, which impairs clot formation and increases the risk of bleeding. In this patient, other hematological tests, such as coagulation time and platelet count, were within normal parameters, which aligns with the diagnosis of Heyde’s syndrome, as the characteristic abnormality does not appear in these basic tests but requires the specific von Willebrand factor profile for detection.

The patient did not require blood transfusions during her hospital stay as she remained hemodynamically stable, and her hemoglobin levels showed no evidence of decline (Hb 8.90 g/dL) nor were there any new episodes of bleeding. This restrictive transfusion strategy aligns with current recommendations, which aim to avoid overtransfusion and its associated risks, such as volume overload, transfusion-related lung injury, increased portal pressure, and potential exacerbation of bleeding.

She was discharged with oral iron supplementation (ferrous sulfate 200 mg every 24 hours) and follow-up appointments in gastroenterology for hemoglobin monitoring and stool occult blood testing. She was also referred to cardiology for a new echocardiogram to continue the protocol for valve replacement, as surgical treatment of aortic stenosis has been shown to reduce gastrointestinal bleeding over prolonged periods, significantly improving quality of life. This is particularly relevant in this patient, who has experienced five episodes of bleeding in the past few years.

## Discussion

The connection between aortic stenosis and gastrointestinal bleeding originates from hemodynamic changes that impair coagulation. In patients with aortic stenosis, the shear stress generated by the narrowed aortic valve fragments the high-molecular-weight multimers (HMWM) of von Willebrand factor through the action of the ADAMTS13 enzyme, resulting in their proteolysis and consequently a deficiency of these multimers. The loss of HMWM compromises platelet adhesion in areas of damaged endothelium, facilitating bleeding, particularly in gastrointestinal angiodysplasias [2].

The clinical manifestations of this syndrome include chronic or intermittent gastrointestinal bleeding, anemia due to blood loss, and symptoms of aortic stenosis, such as dyspnea, chest pain, and syncope. However, some patients may remain asymptomatic, even with significant aortic stenosis. This delays diagnosis, as gastrointestinal bleeding may be attributed to other causes in the absence of evident cardiac symptoms.

Diagnosis of the syndrome requires a multidisciplinary approach, combining clinical history, endoscopy, imaging studies to detect aortic stenosis, and specific von Willebrand factor tests, such as ristocetin cofactor activity and multimer analysis. These tests are crucial to distinguish Heyde’s syndrome from other causes of bleeding, such as hereditary hemorrhagic telangiectasia or anticoagulant use. However, these tests are not always available in all healthcare settings, which can delay diagnosis.

Angiodysplasias that are not actively bleeding may go unnoticed during the endoscopic examination, complicating their diagnosis. They are acquired anomalies whose exact etiology remains poorly understood. While the most common site of intestinal angiodysplasias is the colon, particularly the cecum and ascending colon, due to elevated intermittent wall tension in these areas, these lesions can occur in any part of the gastrointestinal tract [3]. In our patient, the lesion was located in the greater curvature of the stomach. This may be related to altered hemodynamics in the stomach, increased vascular fragility, or specific anatomical features of the patient. However, evidence supporting these factors is limited, highlighting the need for further research.

Although angiodysplasias are the most common source of bleeding in Heyde’s syndrome, other conditions, such as gastric ulcers and diverticulosis, as observed in this case, may exacerbate the risk. This was the case for our patient, who presented with gastritis and a gastric ulcer. This coexistence worsens bleeding due to the fragility and hypervascular nature of these lesions. Inflammatory changes and mucosal erosions contribute to recurrent bleeding episodes. Advances in tools such as capsule endoscopy and angiography have increased sensitivity in detecting GI bleeding sources, including angiodysplasias in the small intestine. Capsule endoscopy complements traditional endoscopy, especially in challenging cases where bleeding sources are difficult to locate. It is particularly useful for identifying subtle lesions in the small intestine, while angiography remains valuable in cases of acute or recurrent bleeding [3].

In the management of Heyde’s syndrome, aortic valve replacement is fundamental to controlling bleeding-related symptoms [4], as it restores von Willebrand factor function and reduces the risk of gastrointestinal bleeding in 80% of patients with angiodysplasia [4,5]. Valve replacement can be achieved through surgery or TAVI (transcatheter aortic valve implantation). The choice between conventional surgery and TAVI depends on factors such as age, comorbidities, and surgical risk scores (EuroSCORE or STS). In older patients with diabetes, chronic kidney disease, or frailty, TAVI is a less invasive and preferred option [6].

In the acute setting, desmopressin or von Willebrand factor concentrates can be used to control bleeding. Desmopressin temporarily increases the release of von Willebrand factor from endothelial cells, improving platelet function. Similarly, von Willebrand factor concentrates can replenish HMWM during episodes of acute bleeding, but their effect is transient and does not address the underlying condition. These therapies are useful as a bridge before definitive valve intervention [7]. The treatment of angiodysplasias through endoscopic therapies, such as cauterization, also allows for bleeding control. Specific interventions such as argon plasma coagulation (APC) or mechanical clipping can manage bleeding from angiodysplasias. APC is preferred over mechanical clipping when lesions are superficial, extensive, or lack a defined bleeding point, as in the case of angiodysplasias distributed over broad mucosal areas. In these scenarios, APC treats diffuse bleeding zones, while clipping is more useful for localized and easily identifiable bleeding points. Thus, the choice between APC and clipping depends primarily on the type, extent, and location of the lesion [8].

Diabetes, hypertension, and dyslipidemia can worsen the prognosis of Heyde’s syndrome by accelerating atherosclerosis and exacerbating aortic stenosis. These conditions also increase vascular fragility, contributing to the development of angiodysplasias and hemorrhagic complications. Similarly, careful management of comorbidities and monitoring anticoagulants or antiplatelet medications are essential. In high-risk Heyde’s syndrome patients with bleeding concerns, anticoagulation should be carefully adjusted, reducing or even temporarily suspending it during acute hemorrhagic episodes. For long-term anticoagulation, using lower-risk medications or reducing antiplatelet therapy (e.g., switching from dual to single therapy) can help minimize additional bleeding. Close collaboration among hematology, gastroenterology, and cardiology is essential to balance thrombotic and hemorrhagic risks, while early valve intervention could reduce the need for intensive long-term anticoagulation [9].

Early valve replacement significantly influences gastrointestinal bleeding outcomes by correcting the underlying coagulopathy. However, determining the optimal timing of this intervention in relation to bleeding episodes is a key question, as it could critically impact clinical outcomes. A prospective study or registry addressing this issue would be highly valuable for guiding clinical decisions in Heyde’s syndrome patients. Additionally, randomized controlled trials specifically comparing the long-term efficacy of TAVI versus SAVR (surgical aortic valve replacement) in this population are needed, evaluating not only bleeding recurrence but also mortality and functional outcomes. Another relevant aspect requiring investigation is understanding the mechanisms behind atypical locations of angiodysplasias and their potential relationship with the hemodynamic changes caused by aortic stenosis. Addressing these areas of uncertainty will optimize the management of Heyde’s syndrome and improve patient outcomes.

## Conclusions

Heyde’s syndrome should be suspected in patients with aortic stenosis and recurrent gastrointestinal bleeding. This case highlights the variability in clinical presentations, including atypical locations like gastric angiodysplasia, exacerbated by conditions such as gastric ulcers. Diagnosis relies on specific von Willebrand factor tests, and management requires a multidisciplinary approach, involving cardiology for valve replacement, gastroenterology for lesion treatment, and hematology for coagulopathy correction. Long-term follow-up is essential to monitor prosthetic valve complications and bleeding risks.

This case underscores the importance of a comprehensive evaluation in patients with cardiovascular comorbidities and recurrent bleeding. Early valve replacement reduces bleeding recurrence and improves quality of life, but further research is needed to optimize diagnosis and treatment. Studies should explore atypical angiodysplasia mechanisms, the impact of comorbidities, and the long-term outcomes of SAVR versus TAVI. Advances in diagnostic tools like capsule endoscopy and enteroscopy can aid early detection and better management of this complex syndrome.
